# Health and Self-Regulation among School-Age Children Experiencing Family Homelessness

**DOI:** 10.3390/children4080070

**Published:** 2017-08-04

**Authors:** Andrew J. Barnes, Theresa L. Lafavor, J. J. Cutuli, Lei Zhang, Charles N. Oberg, Ann S. Masten

**Affiliations:** 1 Developmental-Behavioral Pediatrics, Division of General Pediatrics and Adolescent Health, Department of Pediatrics, University of Minnesota Medical School, Minneapolis, MN 55455, USA; 2 School of Professional Psychology, Pacific University, Hillsboro, OR 97123, USA; tlafavor@pacificu.edu; 3 Department of Psychology, Rutgers University, Camden, NJ 08102, USA; jj.cutuli@rutgers.edu; 4 Clinical and Translational Science Institute, University of Minnesota, Minneapolis, MN 55455, USA; zhangl@umn.edu; 5 Division of Epidemiology and Community Health, University of Minnesota School of Public Health, Minneapolis, MN 55454, USA; oberg001@umn.edu; 6 Institute of Child Development, College of Education and Human Development, University of Minnesota, Minneapolis, MN 55455, USA; amasten@umn.edu

**Keywords:** family homelessness, cognitive functioning, chronic health conditions, middle childhood, child development, resilience, psychosocial risk

## Abstract

Children in homeless families have high levels of adversity and are at risk for behavior problems and chronic health conditions, however little is known about the relationship between cognitive-emotional self-regulation and health among school-aged homeless children. Children (*n* = 86; mean age 10.5) living in shelters were assessed for health, family stress/adversity, emotional-behavioral regulation, nonverbal intellectual abilities, and executive function. Vision problems were the most prevalent health condition, followed by chronic respiratory conditions. Cumulative risk, child executive function, and self-regulation problems in children were uniquely related to child physical health. Homeless children experience problems with cognitive, emotional, and behavioral regulation as well as physical health, occurring in a context of high psychosocial risk. Several aspects of children’s self-regulation predict physical health in 9- to 11-year-old homeless children. Health promotion efforts in homeless families should address individual differences in children’s self-regulation as a resilience factor.

## 1. Introduction

Family homelessness is an ongoing and pernicious problem in the United States, with about 191,325 people in families staying in shelters at some point during 2010 and 191,903 during 2014, almost 60% of whom were children under 18 [1]. Meanwhile, many more children are believed to be homeless using definitions that include those using public shelters, private shelters, doubled-up with friends and family, and other circumstances [2]. Pediatric health professionals and other child health advocates increasingly recognize the deleterious effects that residential instability can have upon children’s optimal development [3]. Homeless youth experience more cumulative and recent-life stress than their impoverished but housed peers [4,5]. Thus, problems in health and well-being associated with child homelessness may result from elevated cumulative risk rather than homelessness itself [6].

Self-regulation is one factor that buffers the effects of cumulative risk and refers broadly to the capacity of individuals to modify their own behavior, thoughts, and feelings; while executive functions (EF) are a neurocognitive aspect of self-regulation that contribute to the voluntary control of attention and behavior to achieve goals [7,8]. Self-regulation/EF can be shaped by early experiences, both positive [9] and negative [10]. Self-regulation/EF develop rapidly during the preschool years, are associated with adaptive social-emotional function, and predict school success, particularly among children at risk due to socioeconomic disadvantage or adversity [11,12]. Competent self-regulation is associated with resilience in a wide range of negative circumstances, including poverty [13,14] and family homelessness [15,16]. On the other hand, self-regulation/EF deficits during childhood predict adult health problems and substance dependence independent of intellectual abilities and social class [17]. It remains unclear how self-regulation/EF and health relate among homeless children during the transition to early adolescence. For example, while observational studies of homeless youth have found higher than expected rates of health problems [3], including asthma and chronic respiratory disease [18,19], prior research has not examined the role of self-regulation, and has relied on single-informant, non-validated measures of children’s health [20,21].

The question of whether self-regulation/EF and psychosocial risk are associated with physical health during middle childhood is important, because self-regulation is especially malleable during this period due to the pre-adolescent peak in prefrontal gray matter and associated neurodevelopmental shifts that accelerate the development of executive function in the preteen years [8]. Thus, if associations between risk, self-regulation/EF, and health are found during middle childhood, then future research and clinical interventions among high-risk populations of children would do well to include self-regulation and health as key measures. Studies of adaptive functioning—i.e., attaining age-appropriate developmental milestones in domains such as cognition and emotional/behavioral control—and asthma or other physical health conditions in low-income and other general populations of children have yielded mixed results, leading to the conclusion that adaptive functioning is more likely to be impaired in severe cases of asthma or very poor physical health [22]. Specifically, young children experiencing homelessness and diagnosed with asthma, compared to those without asthma, had higher parent-reported attention-deficit/hyperactivity symptoms and behavioral problems [23], though this was not observed in a subsequent sample [2], and neither study found differences in EF. However, these studies involved children aged 4–7 and relied solely on parent-report of a physician’s diagnosis of asthma (rather than using validated measures of overall health and/or respiratory symptoms). The current study addresses these limitations by assessing physical health and EF during middle childhood in this population using well-validated measures.

The primary aim of this study was to examine how psychosocial risk, self-regulation, and health are related during middle childhood among youth staying in homeless shelters with their families. We hypothesized that child self-regulation, as reflected by EF from parent reports and direct child testing, would be associated with better child physical health.

## 2. Materials and Methods

### 2.1. Study Design

We conducted a cross-sectional study among children living with adult parents or guardians in two large, urban emergency shelters for homeless families in Minneapolis, MN. Parents were compensated with $30 in gift cards and children were given a small age-appropriate toy for their participation. The Institutional Review Board at the University of Minnesota approved the study (#1007S86252, 7 September 2010).

### 2.2. Participants and Procedure

We recruited 86 nine to eleven-year-old children residing for >48 h in one of two family homeless shelters that house the majority of sheltered families in the metropolitan area. Parents or children who did not speak English were not eligible, nor were siblings of participants. Children provided written assent, and parents provided written consent. In families with multiple parents, extended family members, or other caregivers, only the person primarily responsible for parenting was invited to participate. One family withdrew prior to assessment. The transient nature of shelter life makes it difficult to calculate the denominator of eligible children; to estimate this, we used shelter census figures on the number of families with a 9, 10, or 11-year-old child staying in each of the shelters at mid-week for each full month of data collection. Using this method, we estimated that 71% of eligible families at each shelter participated.

Data was collected in quiet testing suites set up within each shelter. We administered all parent questionnaires via a semi-structured 2-h interview by a trained research associate; a cue card containing visual depictions of scale items was available for each parent-report measure to facilitate understanding. All parent questionnaires were delivered orally given the variability of literacy of parents within this population. Children participated in concurrent one-on-one assessments with another researcher in a neighboring room.

### 2.3. Measures

#### 2.3.1. Predictor: Risk and Adversity

We calculated a Cumulative Risk Index based on existing validated measures to account for differences within the high-risk population of homeless families [24]. This included six items (e.g., child’s parent died; child was abused) from The Life Time Events Questionnaire-Child Version (LTE-C), one item (perceived parent stress) from the Life Events Questionnaire (LEQ), and four items (e.g., number of children in the home; parent’s education level) from a socio-demographic questionnaire. The index was intended to capture the high-risk ends of these scales, which include both discrete and continuous items. For continuous items, dichotomous variables were created using predetermined cutoffs (e.g., four or more children in the home = 1, fewer than four children = 0) [4,25].

#### 2.3.2. Predictor: Self-Regulation

Parents were administered the Behavior Rating Inventory of Executive Function-Parent Report (BRIEF) which has high internal validity and test-retest reliability for children aged 5–18 years [26]. The BRIEF is an 86-item questionnaire that provides a composite total score of EF problems as well as two broader indices of Behavioral Regulation and Metacognition problems. Parents rate their child’s behavior over the past six months on a 3-point scale ranging from “Never” to “Often”. Higher scores represent worse child self-regulation problems.

Parents reported their children’s Emotional Control using the Social Problems scale from the Child Behavior Checklist [27], the Global Emotions scale from the Conner’s Parent Rating Scale [28], and the Emotional Control scale from the BRIEF. These scores were standardized and reverse-coded as needed to create an overall composite that showed high internal consistency (Cronbach’s α = 0.85). Higher scores on this variable indicated better emotional control.

Children were administered two computerized tasks to examine specific cognitive processes associated with EF and behavioral self-regulation. The Color/Word interference task measured task cognitive flexibility and inhibitory control (“shifting and inhibitory control”), with lower scores indicating more-efficient skills [29]. In the first non-interference condition, the participant named a series of color blocks (e.g., blue, green, and red) presented on the screen. In the second non-interference condition, the participant read a series of words printed on the screen in black ink (e.g., “blue”, “green”, “red”). In the interference condition, the participant had to name the color of the word as printed and ignore the printed word, which is incongruent with the color of the print (e.g., the word “red” is printed in blue). The number of errors were computed and converted to scaled scores for the interference condition; higher scores thus indicate worse inhibitory control and shifting. The Tower of London task measured planning and self-monitoring, with higher scores indicating more-efficient skills [30]. This task requires the participant to respond to increasingly complex puzzles by moving objects on three pegs, while adhering to specific rules about legal moves (e.g., can only move one object at a time), in order to replicate the target pattern. The number of moves required to successfully complete the puzzle increases with subsequent trials from one to five. The more difficult puzzles require more planning. The total number of problems completed, out of the 15 possible problems, in the minimum number of moves was computed for each participant; thus, higher scores indicate better planning and self-monitoring.

#### 2.3.3. Predictor: Intellectual Function

Children were given the Matrix Reasoning subtest from the Wechsler Abbreviated Scale of Intelligence (WASI) [31], in which participants identify a missing item from a set of choices to complete a picture or puzzle, to assess nonverbal skills (perceptual reasoning) and general intellectual abilities. This subtest was chosen to avoid confounding introduced by verbally-mediated tasks such as vocabulary among our homeless and highly mobile/disadvantaged sample. Raw scores were converted to *T*-scores that are standardized for age and gender, with higher scores indicating better intellectual function.

#### 2.3.4. Outcome: Health

Parent-reported child physical health problems and their impact were assessed using the MacArthur Health and Behavior Questionnaire for Late Childhood and Adolescence-Parent form (HBQ-P) [32] via the Physical Health Problems Index (PHPI), a composite scaled score derived from the mean of two percentile-ranked subscales (r = 0.30): Chronic Medical Conditions (scored as the sum of 22 possible items; Cronbach’s α = 0.57) and Global Physical Health (scored as a mean of 5 items relating to health impairments/limitations; Cronbach’s α = 0.74). We assessed child-reported health via respiratory symptom severity, given a large number of prior studies on the high prevalence of chronic respiratory conditions in this population, using the Brief Pediatric Asthma Screen Plus [33] adapted for this study as a self-report scale (scored as the sum of five yes/no items; Cronbach’s α = 0.63).

### 2.4. Statistical Analysis

Data were first examined for distribution, no variables fell beyond three standard deviations (SD) from means. A review of scatter plots revealed no outliers that were artificially changing the relations between predictor and outcome variables, so all data were included in the analyses. The evaluation of, e.g., q-q plots from each regression, revealed no gross deviations from normal distribution in residuals. Spearman’s correlation coefficients were calculated to determine if there were monotonic bivariate associations between the predictor and outcome variables. Separate multivariable linear regression models were used to test hypotheses regarding adversity/risk and self-regulation/EF predictors of child-reported respiratory symptoms and parent-reported physical health problems after adjusting for child age, sex, and race. Each predictor was examined separately due to multicollinearity among predictors and limited sample size (see Supplementary Table S1). *p*-values and coefficient estimates (β) with standard errors (SE) are reported. All analyses were carried out using the Statistical Analysis System (v. 9.3; SAS Institute, Cary, NC, USA). All *p*-values are two-sided and evaluated against α = 0.05. We report all results without adjustment for multiple comparisons.

## 3. Results

### 3.1. Demographics

Eighty-six children completed the study. Most primary caregivers were mothers (Table 1; *n* = 86 total parents, 82 mothers and 4 fathers, mean caregiver age = 33.3 years, range = 25–53 years). Sixty-four percent (64%) of children were identified by their parents as African American, 8.1% Native American, 10.5% biracial or multiracial, and 8.1% another race or ethnicity. Thirty-four of the children had been homeless before, with a mean of 1.8 prior times homeless among these 34 youth (range = 1–5; SD = 1.1). Table 2 details participants’ psychosocial adversity that comprised the Cumulative Risk Index.

### 3.2. Health Conditions

**Figure 1:**
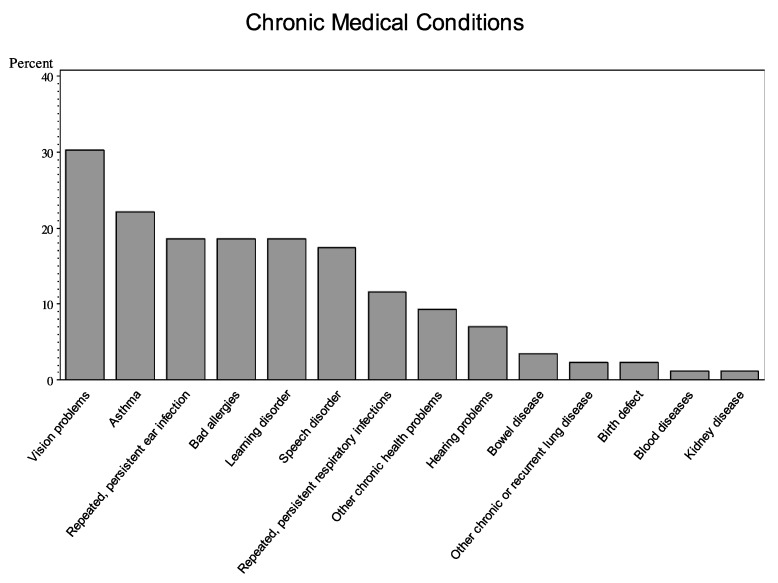
Frequencies of Health Problems (*n* = 86).

A majority (70%) of children had at least one parent-reported chronic health condition. Vision problems were the most prevalent single chronic condition (Figure 1). Children with chronic respiratory conditions made up the next largest group, with 33% of the sample reporting at least one (asthma, severe allergies, persistent respiratory infections, and/or “other chronic/recurrent lung disease”). Developmental disorders were also common (27% reporting speech and/or learning disorders).

### 3.3. Associations Between Child Characteristics and Health

Child intellectual function (IQ) was not significantly associated with age, sex, cumulative risk, or health measures, but was associated with direct tests of child EF and inversely associated with parent-reported child metacognition problems. Age was associated with planning/self-monitoring but not inhibitory control. Parent reports and child measures of EF were not correlated with one another. Child-reported respiratory symptoms were not associated with parent-reported chronic medical conditions (r = 0.14, *p* = 0.213). Child-parent agreement regarding whether or not the child had asthma was moderate (kappa = 0.60). Parent-reported global physical health was correlated with children’s reports of their respiratory symptoms (r = 0.37, *p* = 0.001). (Bivariate correlations between all predictor and outcome variables, along with two-sample t-tests on mean gender differences for each variable, are detailed in Supplementary Table S1).

### 3.4. Self-Regulation and Health

Multivariate regressions showed positive associations between health status and most indicators of self-regulation. Significant predictors of child-reported respiratory symptoms, adjusting for child age, sex, and race, included cumulative risk as well as self-regulation as defined by low child EF performance (shifting/inhibitory control), parent-reported child EF problems, parent-reported child behavioral regulation problems, and low emotional control. Parent-reported child metacognition problems, child nonverbal IQ, and child task planning/self-monitoring skills did not show unique predictive effects (Table 3). Similarly, significant predictors of parent-reported child physical health problems included cumulative risk, low child EF (shifting/inhibitory control), parent-reported child executive function problems, parent-reported child behavioral regulation problems, parent-reported child metacognition problems, and emotional control.

## 4. Discussion

Among this cohort of 9- to 11-year-old children staying in a shelter for homeless families, the mean levels of self-regulation/EF problems as measured by parent report with the BRIEF were overall at or near population-based norms. Nonetheless, those with worse reported health evidenced lower levels on some aspects of self-regulation/EF, consistent with hypotheses based on studies with adults [19,34]. Inhibitory and emotional control had more overlap with children’s physical health problems than other aspects of EF, such as planning and self-monitoring. The strengths of our study include validated measures of self-reported and parent-reported child health; direct measures of child EF; and a high response rate in a hard-to-reach population. Our findings, which take into account multiple aspects of child physical health such as chronicity and severity, contrast with results from younger homeless children that showed no association between directly tested EF and parent-reported asthma (without taking note of disease chronicity or severity) [2,23]. One implication of these apparently discrepant findings is that chronic disease severity should be taken into account when describing functional outcomes in children; for example, asthma severity is associated with worsening behavioral problems [22]. Another implication is that the impact of cumulative risk on EF and EF-related outcomes, such as behavioral adjustment, may vary by developmental stage [13].

There are multiple explanations as to why child health would be related to cognitive and adaptive functioning. These include unidirectional or bidirectional effects of health and adaptive function on one another, and shared causal pathways that might account for problems in multiple domains of biological and social function in development [7,22,34,35,36]. Health problems could interfere with psychosocial development, for example due to children missing school or not feeling well. Treatments for health problems, such as systemic corticosteroids for asthma, could affect cognition via neural pathways implicated in self-regulation. Stress-reactive systems (such as altered hypothalamic-pituitary axis, cardiac, and immune function) that are altered by chronic physical disease and chronic stress could impact, or be impacted by, self-regulation. Low self-regulation and/or high stress reactivity might in turn negatively impact health-related behaviors and the self-management of chronic health conditions. Poor emotional adjustment to illness could be physiologically stressful to children, directly aggravating health symptoms. Risky environments (physically or emotionally hazardous) could pose health and socioemotional challenges. Future work should strive to disentangle the myriad influences that contribute to the association between adaptive functioning and health.

Additional considerations may help explain the observed associations between EF and health during middle childhood, compared to prior work with younger children, in the context of homelessness [22,23]. First, executive functioning changes and develops rapidly during early childhood [36], and its links to physical health might not emerge until middle childhood. Children in homeless families are likely to experience chronic poverty even though their episodes of homelessness are likely to be relatively brief [37]. Exposure to chronic stress and overwhelming adversity could differentially impact self-regulatory development depending on neuroplasticity and timing of exposure [8]. A heightened vulnerability to risk might thus differentially accrue to children who have adapted to survive in a chronically stressful environment [38]. Additionally, cascade effects [34,39] could occur, in which, for example, chronic toxic stress and its correlates lead to altered autonomic-neurohumoral regulation and cognitive function during early childhood, creating allostatic load [40] that predisposes to problems with health and emotional-behavioral competence that are associated with chronic mental and/or physical health conditions during middle childhood [41].

These results imply an overlap between individual differences in the impact of stress upon children’s self-control and health. This highlights the opportunity for clinical monitoring of, and intervention upon, these possibly bidirectional influences. Child health professionals are well positioned to identify not only risk factors such as toxic stress [42] and family homelessness, but also resilience factors such as self-regulation and high-quality parenting. Furthermore, primary care can be an ideal venue in which to intervene and build protective influences in children’s lives [43,44,45].

Our study is limited by its cross-sectional design. We are thus unable to infer causality or draw conclusions about the longitudinal impact of homelessness upon children’s health and self-regulation. Our small sample limited the statistical power for analyses to test (for example) whether self-regulation might mediate the association between risk and health, or whether the chronicity of homelessness moderates the associations we found. Also, we did not measure all covariates that would be of developmental and health relevance, such as prenatal exposure to alcohol or other drugs. We used standardized measures of health that, while valid and reliable for diverse groups, may fail to detect clinical or sub-clinical conditions in a very high-risk population such as ours. A better understanding of how adversity impacts such measurement would allow researchers to better identify processes vulnerable to context, and to develop intervention and prevention approaches to target specific skills. The results of the current study may not be generalizable; because demographic data on family homelessness and children in emergency shelters is not collected in any systematic manner anywhere in the U.S., we have no data to lend any support to any claims that the homeless population in this metro area is different from, or similar to, any other. However, our study had high ecological validity since assessments were conducted in the shelters where the children and families resided, representing a group of children experiencing acute stress in the form of current homelessness. While there is no direct comparison group in the current study, the use of standardized measures allows for comparisons across normative samples by age and gender. Previous research has established the unique and cumulative effect of homelessness compared to stably housed, poor families, which further supports research within this population regardless of available comparison groups.

## 5. Conclusions

Our findings suggest that problems with some aspects of self-regulation and health co-occur among homeless school-aged children, highlighting the need for longitudinal research to clarify processes that link these problems. The results also suggest that educational and health care policies may need to support systems that protect children’s cognitive-emotional development regardless of housing status. In addition, it may be important for health promotion efforts with homeless families to assess individual differences in children’s cognitive-affective function, including their abilities to inhibit their impulses and control their behaviors. Specifically, clinicians and educators need to understand the relationship between health and self-regulation, especially among high-risk youth, and provide accurate assessments across markers of development and settings. Pre-adolescent homeless children’s impressions about their physical health differ from their parents’, indicating the need for multi-informant approaches to patient-reported outcomes in this population. Appropriate resources, referrals, and interventions are needed within communities to address health and developmental discrepancies when identified. Finally, it will be crucial to evaluate intervention and prevention programs designed to boost health and self-regulation skills, not only to promote resilience among very high-risk youth, but also to test causal theories on the co-occurrence of self-regulation and health problems in youth from residentially unstable families.

## Figures and Tables

**Table 1:** Distribution of Children’s Demographics, Risk, Self-Regulation, and Health (*n* = 86).

	*n* (%)	Range	Mean (SD)
**Covariates**			
Male	46 (53)		
Race			
African American	55 (64)		
Other	27 (33)		
Age		9.1–11.9	10.5 (0.81)
**Predictors**			
Adversity: Cumulative Risk Index		0–8	4.0 (1.8)
Intellectual function: WASI Matrix Reasoning IQ (*T*-score)		24–66	46.1 (10.6)
Self-Regulation: Emotional Control Composite (*Z*-score)		−1.02–2.0	0.0 (0.73)
Self-Regulation: Shifting/Inhibitory Control Task (scaled score)		1–17	9.5 (3.1)
Self-Regulation: Planning/Self-Monitoring Task (raw score)		1–13	7.5 (2.5)
Self-Regulation: BRIEF total (*T*-score)		35.2–84	50.7 (11.4)
Self-Regulation: BRIEF Behavioral Regulation Index (*T*-score)		36–92	52.0 (14,5)
Self-Regulation BRIEF Metacognition Index (*T*-score)		31–89	50.5 (13.2)
**Health Outcomes**			
Physical Health Problems Index		0.16–0.95	0.51 (0.23)
Chronic Medical Conditions		0–7	1.63 (1.69)
Global Physical Health		0–2.6	0.64 (0.62)
Child-reported Respiratory Symptom Score		0–5	2.0 (1.5)

WASI: Wechsler Abbreviated Scale of Intelligence; IQ: Intelligence Quotient; BRIEF: Behavior Rating Inventory of Executive Function; SD: Standard Deviation.

**Table 2:** Adverse Childhood Experiences and Sociodemographic Variables Included in Cumulative Risk Index.

Risk Factors	*n* (%)
Single parent household	71 (83)
Presence of four or more children living in the home	43 (50)
The child experienced the divorce of his or her parents	42 (49)
The parent’s perceived level of stress was extremely stressful	42 (49)
Parent under 18 years of age when first child was born	36 (42)
The child saw violence happening to other people	33 (38)
Parent education less than a high school degree	30 (35)
The child saw a parent injured by another person	26 (30)
The child was placed in foster care	12 (14)
The child was the victim of physical or sexual abuse	7 (8)
One of the child’s parents died	5 (6)

**Table 3:** Multivariable Linear Regression Analysis of Predictors of Child Physical Health.

	Physical Health Problems Index (Parent-Reported) β (SE)	Child-Reported Respiratory Symptoms β (SE)
Adversity/Cumulative Risk Index	0.03 (0.02) *	0.3 (0.1) **
**Intellectual Functioning**		
WASI Matrix Reasoning IQ (*T*-score)	−0.002 (0.002)	−0.02 (0.02)
**Self-Regulation**		
Emotional Control Composite (*Z*-score) ^	0.1 (0.3) ***	0.6 (0.2) *
Task Shifting and Inhibitory Control Task Score (scaled score)	−0.02 (0.008) *	−0.1 (0.05) *
Planning and Self-Monitoring Task Score (raw score)	−0.02 (0.01)	−0.1 (0.08)
BRIEF Total (*T*-score) ^	0.006 (0.002) **	0.03 (0.01) *
BRIEF Behavioral Regulation Index (*T*-score) ^	0.005 (0.001) **	0.03 (0.01) *
BRIEF Metacognition Index (*T*-score) ^	0.005 (0.002) *	0.02 (0.01)
**Health**		
Child-reported Respiratory Symptom Score	0.05 (0.02) **	---

Based on separate multivariable linear regressions adjusted for child age, sex, and race. SE: Standard Error; ^ Higher values indicate worse self-regulation; * *p* <0.05; ** *p* <0.01; *** *p* <0.001.

## Supplementary Materials

The following are available online at www.mdpi.com/2227-9067/4/7/70/s1, Table S1: Bivariate Correlations.
