# Supplementary material for: Health and Self-Regulation among School-Age Children Experiencing Family Homelessness
Source: Children (Basel). 2017 Aug 4;4(8):70. doi: 10.3390/children4080070 (PMC5575592; doi:10.3390/children4080070)
Supplement: Supplementary file 1 [file children-04-00070-s001.docx]

|  |  |  | | ***Self-Regulation*** | | | | | | | | | | | | ***Health*** | | | | | | |
| --- | --- | --- | --- | --- | --- | --- | --- | --- | --- | --- | --- | --- | --- | --- | --- | --- | --- | --- | --- | --- | --- | --- |
| **CRI** | **IQ** | | **EC** | | **IC** | | **P/SM** | | **B-T** | | **B-BRI** | | **B-MCI** | | **PHPI** | | **CMC** | | **GPH** | | **RSS†** |  |
| **Gender^** | .44 | .48 | | .80 | | .24 | | .50 | | .54 | | .85 | | .47 | | .86 | | .96 | | .56 | | .05 |
| **Age** | -.07 | .10 | | -.05 | | .00 | | .24* | | -.03 | | -0.01 | | -.01 | | .17 | | .19 | | .08 | | .02 |
| **Cumulative Risk Index (CRI)** | --- | -.18 | | .31** | | -.08 | | -.06 | | .35** | | .42*** | | .30** | | .22* | | .12 | | .23* | | .32** |
| **WASI Matrix Reasoning (IQ)** | --- | --- | | -.07 | | .32** | | .29** | | -.18 | | -.13 | | -.22* | | .00 | | .10 | | -.07 | | -.16 |
| **Emotional Control (EC)** | --- | --- | | --- | | -.07 | | 0.29** | | .65*** | | .78*** | | .59*** | | .36** | | .31** | | .27* | | .34** |
| **Task Shifting and Inhibitory Control (IC)** | --- | --- | | --- | | --- | | 0.28* | | -.13 | | -.10 | | -.16 | | -.20 | | -.10 | | -.19 | | -.26* |
| **Planning and Self-Monitoring (P/SM)** | --- | --- | | --- | | --- | | --- | | -.12 | | -.05 | | -.12 | | -.14 | | -.1 | | -.1 | | -.13 |
| **BRIEF Total (B-T)** | --- | --- | | --- | | --- | | --- | | --- | | .92*** | | .96*** | | .32** | | .34* | | .15 | | .24* |
| **BRIEF Behavioral Regulation Index (B-BRI)** | --- | --- | | --- | | --- | | --- | | --- | | --- | | .83*** | | .32*** | | .30** | | .22* | | .31** |
| **BRIEF Metacognition Index (B-MCI)** | --- | --- | | --- | | --- | | --- | | --- | | --- | | --- | | .26* | | .31** | | .08 | | .22 |
| **Physical Health Problems Index (PHPI)** | --- | --- | | --- | | --- | | --- | | --- | | --- | | --- | | --- | | .79*** | | -.13 | | .31*** |
| **Chronic Medical Conditions**  **(CMC)** | --- | | --- | | --- | | --- | | --- | | --- | | --- | | --- | | --- | | .30** | | .14 | |
| **Global Physical Health (GPH)** | --- | | --- | | --- | | --- | | --- | | --- | | --- | | --- | | --- | | --- | | .37** | |
| ^ p-values from two sample t-tests of Male vs. Female  † Child-Reported Respiratory Symptom Score; WASI: Wechsler Abbreviated Scale of Intelligence; BRIEF: Behavior Rating Inventory of Executive Function  *** p < .05**  **** p < .01**  ***** p < .001** | | | | | | | | | | | | | | | | | | | | | | |

Supplementary Table 1. Bivariate Correlations.
